# Cell Death and Survival Pathways Involving ATM Protein Kinase

**DOI:** 10.3390/genes12101581

**Published:** 2021-10-07

**Authors:** Toshihiko Aki, Koichi Uemura

**Affiliations:** Department of Forensic Medicine, Graduate School of Medical and Dental Sciences, Tokyo Medical and Dental University, Tokyo 113-8519, Japan; kuemura.legm@tmd.ac.jp

**Keywords:** ATM, apoptosis, necroptosis, ferroptosis, autophagy

## Abstract

Cell death is the ultimate form of cellular dysfunction, and is induced by a wide range of stresses including genotoxic stresses. During genotoxic stress, two opposite cellular reactions, cellular protection through DNA repair and elimination of damaged cells by the induction of cell death, can occur in both separate and simultaneous manners. ATM (ataxia telangiectasia mutated) kinase (hereafter referred to as ATM) is a protein kinase that plays central roles in the induction of cell death during genotoxic stresses. It has long been considered that ATM mediates DNA damage-induced cell death through inducing apoptosis. However, recent research progress in cell death modality is now revealing ATM-dependent cell death pathways that consist of not only apoptosis but also necroptosis, ferroptosis, and dysfunction of autophagy, a cellular survival mechanism. In this short review, we intend to provide a brief outline of cell death mechanisms in which ATM is involved, with emphasis on pathways other than apoptosis.

## 1. Introduction

Cellular genomic DNA is always exposed to the risk of damage caused by ultraviolet light (UV), ionizing irradiation (IR), and exposure to chemicals, what is collectively called genotoxic stress [1,2]. In addition to these extrinsic stresses, the intrinsic generation of reactive oxygen species (ROS), mainly in mitochondria, is another stress leading to injuries in both genomic and mitochondrial DNA [3]. Although the detrimental impacts of ROS include lipid peroxidation [4], protein oxidation [5,6], and oxidative inactivation of enzyme [5,6], it can damage DNA through the formation, for example, of 8-oxoguanine, which is observed ubiquitously even in healthy cells [7,8]. Aberrant DNA replication such as replication fork collapse [9], as well as defects in DNA repair [10], is often associated with DNA damage and is followed by cellular responses to maintain whole body homeostasis. To avoid the accumulation of damaged DNA as well as aberrant DNA replication, cells have a sophisticated system called the DNA damage response (DDR) [11,12]. DNA damage, such as single- and double-stranded DNA breaks (SSBs and DSBs), as well as DNA adduct formation, are recognized by sensor proteins, which initiate the DDR by activating transducer and effector proteins. ATM (ataxia telangiectasia mutated) and ATR (ATM and rad3-related) are the most important transducer proteins [1,13]. While ATM is activated primarily by DSB, ATR is activated by a broader spectrum of stresses including SSB and DSB [1].

ATM activation is governed by the MRN complex, which consists of mitotic recombination 11 (Mre11), Rad50 double strand break repair protein (Rad50), and Nijmegen breakage syndrome 1 (Nbs1), and works as a sensor protein bridging DSBs and ATM [14]. When a DSB is generated in cells, the MRN complex recognizes the ends of DNA breaks and recruits ATM to the ends, where ATM self-activates through autophosphorylation at ser-1981 [15,16]. This autophosphorylation results in the conversion of inactive ATM dimers into active monomers [15]. Autophosphorylated and activated ATM further phosphorylates H2A.X variant histone (H2AX). Ser-139 phosphorylated H2AX (γH2AX) spreads the DNA damage response along the chromatin [17]. It should be noted that ATM-independent generation of γH2AX has also been reported [18]. Downstream of the generation of DSBs and subsequent activation of ATM, the most important role of ATM is regulation of cell cycle checkpoint. In addition, there are two cellular responses: DNA repair and cell death. The DNA repair response is further divided into two responses: homologous recombination (HR) and non-homologous end-joining (NHEJ) [2]. Although ATM seems to be directly involved in the HR process [19], DNA-dependent protein kinase (DNA-PK) rather than ATM might be the central molecule involved in NHEJ [2,20]. Nevertheless, massive genotoxic stress surpassing the cellular ability to repair the resulting DNA damage should lead to cell death or the development of cancer.

In this brief review, we intend to provide minimal essential information of fundamental mechanism of cell death for the researchers who are interested in not only ATM but also cell death. Although there are an ever-growing number of cell death modes, we pick up apoptosis, necroptosis, and ferroptosis; ATM is suggested to be involved in these modes of cell death.

## 2. Role of ATM in Apoptosis

Apoptosis should be the most extensively studied form of cell death induced by DNA damage. Since there are numerous review articles describing various aspects of apoptosis (for example, [21,22]), we briefly summarized only fundamental mechanisms of apoptosis.

**Figure 1:**
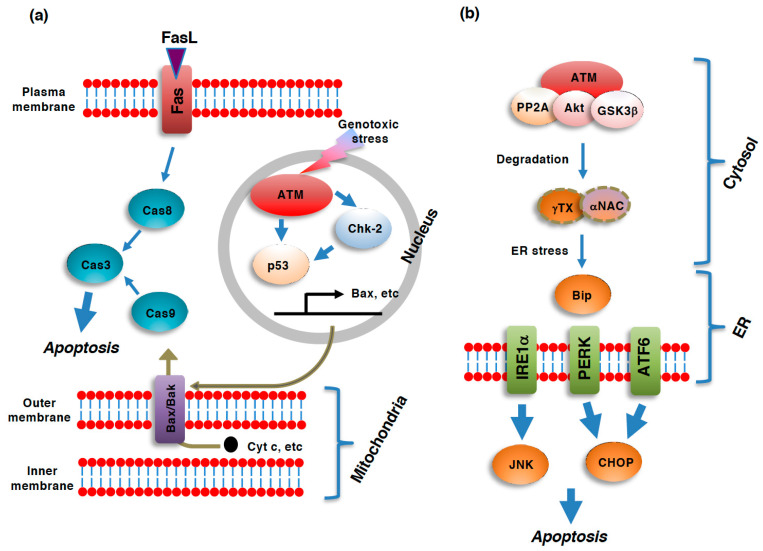
Role of ATM in various pathways of apoptosis. Pathways of apoptosis and its regulation by ataxia telangiectasia mutated kinase (ATM). (**a**) Extrinsic apoptosis and intrinsic apoptosis are two major pathways of apoptosis. In the intrinsic pathway, a panel of mitochondrial intermembrane space (IMS)-resident proteins, such as cytochrome c (cyt c) and Smac/DIABLO (direct IAP binding protein with low pI) [36,37], are released into the cytoplasm in response to the dysregulation of mitochondrial function, including a loss of mitochondrial outer membrane potential (ΔΦm), an increase in mitochondrial outer membrane permeability (MOMP), or the generation of reactive oxygen species (ROS), through pores in the mitochondrial outer membrane composed of oligomers of bax and bak [38,39] to activate caspase-9 [40,41]. Caspase-9 then activates caspase-3 to execute the downstream events of the entire apoptotic process. In contrast to the intrinsic pathway, the extrinsic pathway is initiated by the ligation of so-called death ligands, such as FasL, to cell surface receptors [42,43,44]. Although the ligation of the receptors to death ligands typically results in caspase-8 activation, this caspase also leads to the activation of caspase-3 [45,46]. The DNA damage response (DDR)-induced activation of ATM results in the subsequent activation of p53 via direct phosphorylation as well as checkpoint kinase 2 (Chk2)-mediated phosphorylation. p53 transactivates bax gene expression, which facilitates the mitochondrial pathway of apoptosis. (**b**) ER stress-induced unfolded protein response (UPR) also leads to apoptosis. UPR is executed via three pathways: the inositol-requiring enzyme1α (IRE1α), PKR-like ER kinase (PERK), and activating transcription factor6 (ATF6) pathways. c-Jun N-terminus kinase (JNK) and C/EBP homologues protein (CHOP) serve as the mediators of apoptosis. All three of these pathways are regulated by the ER resident chaperon, binding immunoglobulin protein (BiP). ATM facilitates ER stress and subsequent apoptosis through the protein phosphatase 2A (PP2A)/Akt/ glycogen synthase 3β (GSK-3β)-dependent degradation of nascent polypeptide-associated complex α-subunit (αNAC)/ γ-taxilin (γTX). In this case, cytoplasmic ATM serves as a platform for the activation of the PP2A/Akt/GSK-3β axis [47].

Mechanistically, apoptosis is regulated, as well as executed, through a cascade of the activation of proteases known as caspases [23,24]. Caspases can be categorized into two groups: initiator caspases (caspase-2, -8, -9, -10), which are involved in the initiation of apoptosis, and executioner caspases (caspase-3, -6, -7), which are activated by initiator caspases and involved in the executional processes of apoptosis [23]. Executioner caspases are involved in the degradation of cellular molecules essential for cell survival. Undoubtedly, p53 is the most characterized protein involved in DNA-damage induced apoptosis [25] (Figure 1). This is also the case of apoptosis proceeding through the ATM-dependent manner [26]. ATM is involved in the DDR-induced activation of the intrinsic apoptotic pathway mainly through p53. ATM and its downstream effector, checkpoint kinase 2 (Chk2), can phosphorylate p53 at ser-15 and ser-20, respectively, which stabilizes p53 by disrupting the binding of E3 ubiquitin ligases and subsequently protecting it from proteolytic degradation by the 26S proteasome [25,27,28,29]. In addition to ser-15 and ser-20, the phosphorylation of p53 at ser-46 during DDR has also been reported to play an important role in DNA damage-induced cell death [26]. Phosphorylated and subsequently stabilized p53 induces the expressions of a panel of pro-apoptotic genes, such as bax, thereby facilitating the mitochondrial apoptotic pathway [30,31]. In addition to its role as a transcription factor, p53 has also been reported to facilitate mitochondrial pathway of apoptosis by recruiting bax to mitochondria [32]. It should be noted that ATM can induce apoptosis through various axes other than p53-dependent axis, such as p73-dependent activation of mitochondrial pathway of apoptosis (reviewed in [2]). 

DNA damaging stimuli such as UV, IR and exposure to chemicals can also damage cellular proteins. Therefore, DNA damage is often associated with ER stress, which elicits the subsequent unfolded protein response (UPR) in stressed cells [33]. Like DDR, UPR can result not only in cellular protection by facilitating the degradation of misfolded proteins, but also in the induction of apoptosis when cellular stresses surpass the cellular repair capacity [34,35]. ER stress is sensed by a chaperon protein that resides in the luminal space of the ER, binding immunoglobulin protein (BiP), and is relayed to the three forms of UPR pathway: the inositol-requiring enzyme1α (IRE1α), PKR-like ER kinase (PERK), and activating transcription factor6 (ATF6) pathways (Figure 1). IRE1α and PERK are kinases located at the ER membrane, while ATF6 is a transcriptional activator that translocates from the ER to the Golgi apparatus where ATF6 is cleaved to become its active form. Pro-apoptotic c-jun N-terminus kinase is the main executioner of IRE1α pathway, while C/EBP homologous protein serves as the main mediator of both PERK- and ATF6-depednent apoptosis [34].

Cytoplasmic ATM has recently been suggested to be involved in the UPR upstream of BiP. ATM serves as a platform supporting the protein phosphatase 2A (PP2A)-dependent dephosphorylation of Akt and subsequent activation of glycogen synthase 3β (GSK-3β) [47]. The kinase activity of ATM seems to be unnecessary for this protein complex to work as the mediator of ATM-dependent cell death; ATM works as a bridging protein connecting Akt with PP2A for inactivation via PP2A-depedent dephosphorylation [47]. This axis of protein phosphorylation/dephosphorylation events leads to the degradation of the nascent polypeptide-associated complex α-subunit (αNAC) and γ-taxilin (γTX), both of which are required for the proper transport of nascent polypeptides from ribosomes into the ER [48]. In accordance with the notion that this pathway acts upstream of BiP, the inhibition of ATM results in the activation of UPR and subsequent apoptotic cell death [40]. 

In stark contrast to necrosis, apoptosis is not associated with plasma membrane rupture. Therefore, unlike necrosis, apoptotic cells do not extrude their cellular contents and do not elicit an immune reaction by neighboring cells; cells undergoing apoptosis are rapidly eliminated through phagocytosis by macrophages [49]. In contrast, necrosis is often associated with tissue inflammation due to the release of the cellular contents into the extracellular milieu [50]. Given the importance of inflammation in the pathology of many diseases, this difference between apoptosis and necrosis gives necrosis certain significance; we should consider the possible involvement of necrosis in the pathology of diseases of interest, especially when the disease is accompanied by non-negligible levels of inflammation. Although apoptosis was considered to be the only mechanism of ATM-induced cell death executed in a regulated manner, recent research advances have been revealing examples of ATM-induced regulated from of necrosis, which are described in the following sections.

## 3. Role of ATM in Necroptosis

Necroptosis is one of the first forms of regulated necrosis found to be executed in a regulated manner both mechanically and genetically [51]. Necroptosis was discovered as an alternative form of cell death that was still observed in the L929 mouse fibroblast-like cell line in which apoptosis was blocked by the caspase inhibitor zVAD-FMK [52]. Although the role of necroptosis in the homeostasis of the human body has not been elucidated, necroptosis is assumed as an alternative form of death mechanism of cells in which apoptosis is suppressed. Necroptosis was also identified as a form of ischemic brain cell death that can be blocked by the small molecule inhibitor necrostatin-1 (nec-1) [53]. Later, receptor-interacting kinase-1 (RIP1) was identified as the target of nec-1 [54]. Canonical necroptosis, for example the stimulation of immune cells such as monocytes/macrophages by pro-inflammatory cytokines such as TNFα, is executed through the formation of a complex between RIP1 and RIP3 downstream of the TNF receptor, and the subsequent activation of mixed lineage kinase domain-like (MLKL), which translocates to the plasma membrane as a trimer and is believed to be involved in the formation of the plasma membrane pores required for the rupture of the plasma membrane during necrosis [55,56,57,58].

**Figure 2:**
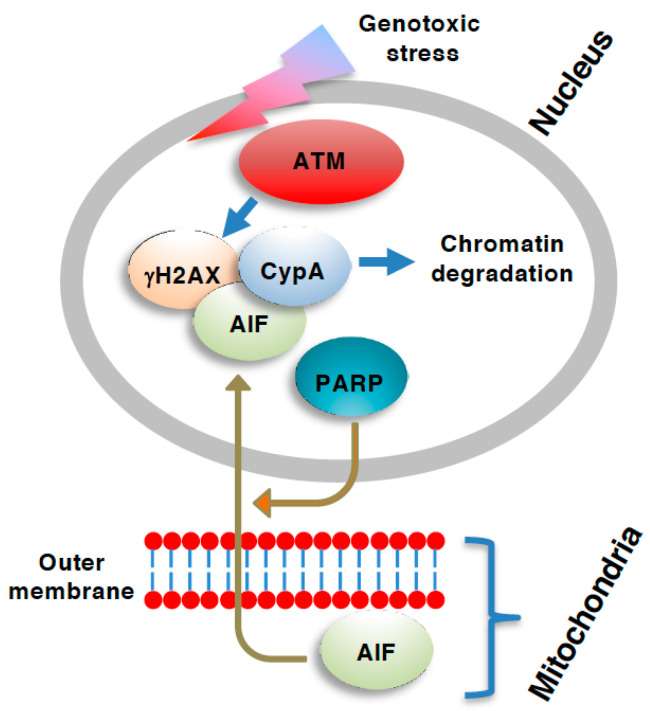
Role of ATM necroptosis. The apoptosis-inducing factor (AIF)-dependent caspase-independent form of necroptosis [or parthanatos when the AIF-dependent cell death is also dependent on poly(ADP-ribose) polymerase (PARP)] involves the translocation of AIF from the mitochondria into the nucleus [69]. Nuclear AIF is complexed with ser-139 phosphorylated H2A.X variant histone (γH2AX) and assists the digestion of chromatin via cyclophilin A (CypA) [64]. The generation of γH2AX is mediated by ATM, and, therefore, ATM is required for the AIF-dependent type of necroptosis.

In addition to the RIP1/RIP3/MLKL axis of necroptosis, other necroptosis axes have been reported, for example, apoptosis-inducing factor (AIF)-dependent and caspase-independent cell death [59] (Figure 2). AIF, which was first implicated in apoptosis but later found to be also involved in necrosis, is a NADH-dependent oxidoreductase that resides in mitochondria in healthy cells, but is cleaved by calpain and translocates into the nucleus in a truncated form (tAIF) where it participates in the degradation of chromosomal DNA at the boundaries of nucleosomes [60,61,62,63,64]. Poly(ADP-ribose) polymerase (PARP) is also implicated in AIF-dependent necroptosis. Upon DNA damage caused by DNA alkylating reagents such as N-methyl-N’-nitro-N-nitrosoguanidine (MNNG), PARP, which is involved in DNA repair, is overactivated resulting in the generation of excess poly(ADP-ribose) (PAR). Excess activation of PARP leads to necrosis though the depletion of cellular NAD+ as well as ATP [65]. Furthermore, PARP activation upon DNA damage and the resultant generation of PAR facilitates the translocation of AIF from mitochondria into the nucleus [66]. This type of necroptosis mediated by the PARP-AIF axis is also called parthanatos [67]. Although there is less information about the possible crosstalk between the RIP1/RIP3/MLKL-dependent and AIF-dependent axes of necroptosis, one report has indicated that hydrogen peroxide elicits necrosis in certain cell types in a RIP1/RIP3/PARP/AIF-dependent manner [68].

As described, AIF-dependent type of necroptosis (or parthanatos) is elicited by DNA damaging reagents and involves DNA degradation, implicating possible involvement of ATM in this mode of cell death. Indeed, Baritaud et al. have reported that ATM is required for MNNG-induced AIF-dependent necroptosis [69]. During this type of necroptosis, the ATM-dependent generation of γH2AX, which is often observed during DNA damage and has been proved to be essential in MNNG-induced necroptosis, plays an essential role in the cell death. ATM forms a complex with γH2AX and cyclophilin A, which can degrade DNA when assisted by AIF [70]. AIF-dependent necroptosis is implicated in a variety of pathologies including ischemic injuries, neurodegeneration such as Alzheimer’s as well as Parkinson’s disease, and prostate cancer [71]. Thus, it might be possible to regulate AIF-dependent necroptosis though modulating ATM.

## 4. Role of ATM in Ferroptosis

**Figure 3:**
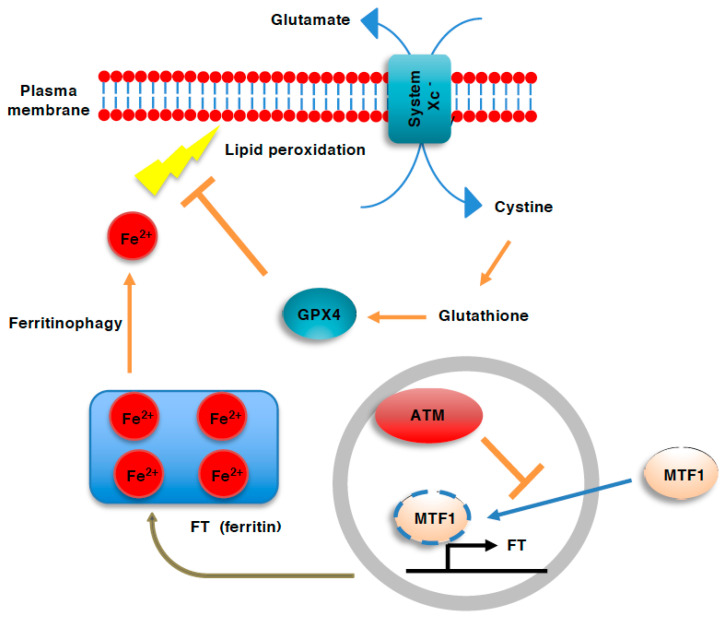
Role of ATM in ferroptosis. Ferroptosis involves plasma membrane damage that results from the iron-dependent generation of reactive oxygen species (ROS) and resulting peroxidation of lipids. Iron is stored in cells as a complex with ferritin (FT). During ferroptosis, FT is degraded to release cellular labile iron through autophagy (ferritinophagy). ATM deficiency activates metal regulatory transcription factor1 (MTF1), which induces ferritin gene expression, thereby enhancing iron storage and preventing ferroptosis.

Ferroptosis is an iron-dependent form of cell death characterized by lipid peroxidation and subsequent damage to the plasma membrane, although the mechanism connecting lipid peroxidation to plasma membrane rupture has not been elucidated [72,73]. Ferrous iron (Fe^2+^) is involved in the formation of ROS though the Fenton reaction, which leads to the subsequent peroxidation of phospholipids (Figure 3). Therefore, ferroptosis depends on the presence of ferrous iron [74].

Glutathione peroxidase4 (GPX4), a unique member of the glutathione-dependent peroxidase family that has the ability to reduce peroxidized lipids, has been shown to play a central role in the prevention of ferroptosis in healthy cells [75,76]. System Xc^-^, the glutamate—cystine antiporter involved in the transport of cystine, a precursor of glutathione, from the extracellular environment into cells, is also important for the prevention of ferroptosis. 

An unexpected role of ATM in ferroptosis has been revealed by Chen et al. [77] who, using the siRNA-based screening of kinases involved in the execution of ferroptosis, identified ATM as an essential kinase for ferroptosis [77]. They also revealed that ATM regulates ferroptosis positively by facilitating iron metabolism. ATM inhibition not only increases both the heavy and light chains of ferritin, which are involved in the cellular storage of iron, but also increases ferroportin, which is involved in the export of iron [77]. Thus, ATM is involved in the increase in the levels of cellular labile iron required for ferroptosis. This ATM regulation of iron storage proteins seems to be mediated by metal regulatory transcription factor1 (MTF1), which is involved in the cellular storage of iron through inducing ferritin. ATM facilitates ferroptosis through inactivating MTF1, thereby facilitating the elevation of cellular labile iron pools required for ferroptosis. Nevertheless, whether ATM regulates MTF1 directly or indirectly remains to be examined [77]. The essential role of ATM in ferroptosis appears to be consistent with recent reports that have identified lipid peroxidation and ferroptosis as cellular responses to ionizing radiation, a typical inducer of DDB [78]. 

## 5. Role of ATM in Autophagy

**Figure 4:**
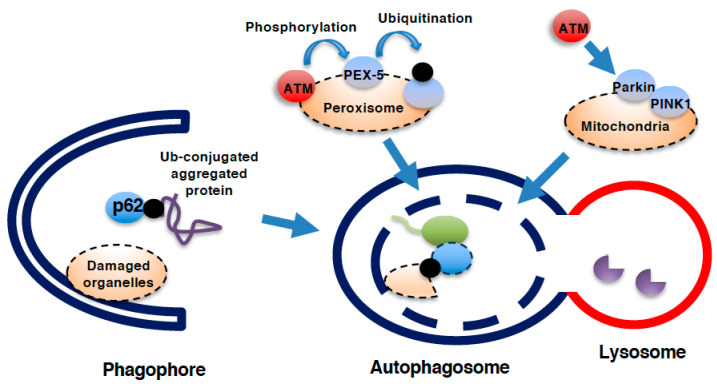
Role of ATM in autophagy. Process of autophagy. During the formation of the phagophore and further creation of the autophagosome, microtubule-associated protein light chain 3 (LC3) is cleaved and conjugates with phosphatidylethanolamine (PE) to insert itself in the autophagosomal membrane. Ubiquitinated (Ub-) protein aggregates that cannot be degraded by the proteasome are delivered into the autophagosome via the binding of p62 to both LC3 and Ub-proteins. Damaged organelles, such as mitochondria, peroxisomes, and even the nucleus and lysosomes, can be delivered into autophagosomes for lysosomal degradation. Autophagosomes fuse with lysosomes for the degradation of interior proteins by lysosomal hydrolases. ATM can facilitate mitochondrial autophagy (mitophagy) as well as peroxisomal autophagy (pexophagy). During pexophagy, ATM phosphorylates the peroxisomal membranous protein PEX-5 to facilitate its ubiquitination and subsequent binding to p62. During mitophagy, ATM activates PTEN-inducible kinase1 (PINK1)/parkin system.

Autophagy is a cellular degradation system that contributes to cell survival by recycling biomaterials under nutrient starved conditions [79,80]. Autophagy also contributes to cellular homeostasis by eliminating damaged proteins, which are harmful when they accumulate in cells. Mechanically, autophagy is initiated via the formation of a phagophore/isolation membrane, a cellular structure made up of membrane lipids (Figure 4). After expansion, nucleation, and closure of the phagophore, a double membrane structure, called an autophagosome, is created in which cellular proteins or organelles are included. During the formation of autophagosome, microtubule-associated protein light chain 3 (LC3) is cleaved, conjugated with phosphatidylethanolamine, and inserted into the autophagosomal membranes [81]. Not only soluble but also aggregated proteins can be sorted into the interior space of autophagosomes. For example, ubiquitin-conjugated protein aggregates, generated due to the inability of proteasome to digest large protein aggregates, are delivered to autophagosome though p62, which can bind to both ubiquitin and LC3 [81]. The materials inside the autophagosomes are then delivered to the interior of lysosomes for degradation. This step is achieved by the fusion of the outer membrane of the autophagosomes with the lysosomal membrane to produce a fusion structure known as an autolysosome. Syntaxin17 serves as an autophagosomal SNARE protein in this fusion process [82]. As a natural consequence of its role in cellular survival/homeostasis, autophagy is also involved in the regulation of cell death under some circumstances. Although autophagy is a cellular protective system against the accumulation of misfolded proteins as well as nutrient deficiency, the aberrant accumulation of autophagosomes/autolysosomes and subsequent cell death are frequently observed under a variety of circumstances. These cell deaths are collectively known as autophagic cell death, although autophagy itself is disrupted due to, for example, a loss of lysosome activity in most cases of autophagic cell death [83,84].

ATM participates in the regulation of autophagy. For example, it has been reported that mitophagy, which is the process by which damaged mitochondria are degraded through autophagy, is decreased in the thymus of ATM-null mice [85]. ATM seems to activate mitophagy through upregulating PTEN-inducible kinase1 (PINK1)/Parkin system [86,87], which is essential for mitophagy [88]. It has been shown that a loss of ATM leads to the death of neurons, at least in part through the upregulation of autophagy [89]. Pexophagy, which is a form of specified autophagy targeting peroxisomes, is another example in which ATM is involved. ROS generation from peroxisomes can activate ATM through modifying its sulfhydryl groups [90]. ATM activated on peroxisomal membranes phosphorylates ser-141 residue of peroxisomal biogenesis factor-5 (PEX-5), thereby facilitating the ubiqutination of this protein and resultant binding to p62 [90]. This ATM-dependent phosphoprylation of PEX-5 on peroxisomes initiates pexophagy, which should be beneficial for cellular homeostasis. As autophagy is essential for human body homeostasis and its dysregulation participates in the development of numerous diseases [91], this cellular protective process should be important target of ATM to maintain whole body homeostasis.

## 6. Concluding Remarks and Future Perspectives

Recent research progress is now revealing that necroptosis, parthanatos, and ferroptosis must be taken into accounts in the research field of ATM-dependent cell death. Since pathophysiological implications of these cell death are totally different from that of apoptosis, we should reconsider the role of ATM in the pathogenesis of relevant diseases. For example, the role of ATM in ferroptosis might implicate that ATM participates in the pathogenesis of neurodegenerative diseases such as Parkinson’s disease, since ferroptosis is implicated in this disease [92]. There are other forms of regulated cell death such as pyroptosis [93], autosis [94], and methuosis [95], and we should also pay attention to the possible involvement of ATM in these cell deaths. Since the investigation of the ATM-dependent regulation of cell death other than apoptosis has just started, there is a possibility that unexpected roles of ATM may be revealed by the research.

## Data Availability

Not applicable.
